# Prevalence and predictors of chemotherapy-induced peripheral neuropathy among female breast cancer patients undergoing chemotherapy in Lagos

**DOI:** 10.3332/ecancer.2024.1791

**Published:** 2024-11-01

**Authors:** Ademola Oluwatosin Oyekan, Omolara Aminat Fatiregun, Muhammad Habeebu, Ifeanyichukwu Augustine Onyeodi, Adebayo Deborah Adeoluwa

**Affiliations:** 1 Department of Clinical Oncology, Lagos State University Teaching Hospital, Ikeja 101233, Nigeria; 2 Department of Radiotherapy and Oncology, Lagos University Teaching Hospital, Lagos 102215, Nigeria; 3 Department of Community Health and Primary Care, College of Medicine, University of Lagos, Idi-araba, Lagos 101017, Nigeria

**Keywords:** chemotherapy-induced peripheral neuropathy CIPN, breast cancer patients, chemotherapy

## Abstract

**Background:**

Chemotherapy-induced peripheral neuropathy (CIPN) is a major side effect associated with chemotherapy. It can lead to detrimental dose reductions and discontinuation of treatment because of its significant effect, which impairs the quality of life among the surviving population of cancer patients. This study assesses the prevalence and predictors of CIPN among female breast cancer patients receiving chemotherapy at the Lagos University Teaching Hospital and Lagos State University Teaching Hospital (LUTH and LASUTH), respectively.

**Methods:**

261 women with histologically confirmed breast cancer who had just concluded first line chemotherapy were recruited for this study. The relevant data was obtained using a designed/semi-structured questionnaire for patient demographic information, and clinical information was retrieved from the participants’ medical records, CIPN symptoms were collected using the European Organisation for Research and Treatment of Cancer CIPN20 and analysed.

**Results:**

Two hundred and sixty-one female breast cancer patients receiving either neoadjuvant or adjuvant chemotherapy were enrolled in the study. The mean age was 49.98 +/- 11.4 years. 72.9% (183) among the study participants reported symptoms of CIPN at the end of chemotherapy. One hundred and fifty-seven (62.5%) had mild neuropathy, 23(9.2%) had moderate neuropathy and 3 (1.2%) had severe neuropathy. 31.1% (74) of patients at 2 months after completion of chemotherapy still reported symptoms of CIPN. Numbness in both hands and legs was found to be the most common symptom reported by the participants and the majority of the participants experienced mild to moderate symptoms.

**Conclusion:**

The prevalence of CIPN was high at the completion of chemotherapy (72.9%), and there was a significant decline in the prevalence at two months after completion of treatment (31.1%). Numbness was the most commonly reported symptom among the participants and the majority of the participants experienced mild symptoms of peripheral neuropathy.

## Introduction

Breast cancer is the most common type of female cancer in both developed and developing countries. The highest incidence rate of breast cancer among women in Australia (95.5 per 100,000 woman-years), and the lowest rate was seen in south-central Asia (26.2) [1]. An estimated 20 million new cases were proposed to be diagnosed globally and 9.7 million cancer deaths in 2022. Female breast cancer represents 11.6% of all newly diagnosed cancer, globally and it accounts for 6.9% of cancer mortality globally [2].

In Nigeria, it has been reported that there is a rise in the incidence of breast cancer, which may be due to social and behavioural changes in Western lifestyles, reproductive factors, increased awareness and screening [3]. Breast cancer is a widely heterogeneous disease with several histological types that can be considered a non-invasive or invasive disease. Invasive ductal carcinoma is the most common histological type accounting for 70%–80% of all invasive lesions [4]. According to the St Gallen International Breast Cancer Conference in 2011, there are four breast cancer intrinsic subtypes based on the surrogate markers; Oestrogen receptor (ER), Progesterone receptor (PR), Human Epidermal receptor status and Ki-67, they include luminal A (ER+ and/or PR+, Ki67 low and HER2-), luminal B (ER+ and/or PR+, Ki67 high and/or HER2+), HER2-positive (ER-, PR- and HER2+) and triple-negative (ER-, PR- and HER2-). This profiling predicts the pathologic characteristics, eventual response to systemic treatment and overall survival [5, 6].

Surgery and radiotherapy play important roles in the local control of breast cancer while chemotherapy, hormonal therapy and targeted therapy are used as either neoadjuvant or adjuvant when ensuring a systemic control of the disease [7]. The chemotherapy agent or the chemotherapy combination used may significantly impact the tumour response and the patient’s clinical outcome [8].

The increase in the population of survivors of breast cancer as a result of improving health literacy, early diagnosis and proficiency in treatment implies that the frequency of late side effects from treatment either from surgery, radiotherapy or chemotherapy will also increase among these patients. Hence, the rise in the prevalence of chemotherapy-induced peripheral neuropathy (CIPN) in breast cancer patients [9].

CIPN is a functional impairment of sensory, motor, or autonomic nerves caused by cancer chemotherapy, resulting in peripheral nervous signs or symptoms; and it is termed ‘persistent’ if it lasts for more than 14 days [10]. It is capable of producing serious neurological impairments and neuropathy; this can lead to detrimental dose reductions and discontinuation of treatment. CIPN when significant can impair the quality of life of patients who survive cancer [11]. The antineoplastic agents known to be involved include platinum-based agents, vinca alkaloids, epothilones, taxanes, proteasome inhibitors and immunomodulatory drugs [12, 13]. These agents have varying mechanisms of causing damage to neurons including oxidative stress, axon degeneration, altered calcium homeostasis, apoptotic mechanisms and membrane remodelling, additionally; immune processes and neuroinflammation [14].

CIPN is a major concern for many physicians, though often overlooked by some. Due to the lack of established treatments, it tends to be underreported and inadequately managed. Currently, duloxetine, a serotonin-norepinephrine reuptake inhibitor is the only proven medication known to ameliorate the symptoms associated with CIPN but it is currently unavailable and more often unaffordable by the majority of the patients in our environment [15]. The prevalence, pathophysiology, co-morbidities, chemotherapy agents that increased the risk of occurrence and preventive and treatment methods have all been studied in cancer patients in different centres across the world including people of all races but very few of them have been done in this environment [11–13, 16–20]. The case of a 28-year-old man with Hodgkin lymphoma, who developed severe neurotoxicity from vinca alkaloid chemotherapy is the only available local study that was reported on CIPN in Nigeria [21]. Hence, there are no adequate studies done on CIPN in this environment. This study assesses the prevalence and predictors of CIPN among patients with breast cancer undergoing chemotherapy. This study will also serve as the foundation for further studies especially when exploring the possible treatment options for CIPN in this environment.

## Materials and methods

### Study site

This two-centred study was carried out in the leading referral tertiary hospitals in Lagos; the Radiotherapy and Oncology department of the Lagos University Teaching Hospital, and the Oncology unit of the Lagos State University Teaching Hospital. They are the main referral centres for other government-owned general hospitals and private hospitals in the state and its environment. Lagos University Teaching Hospital and Lagos State University Teaching Hospital (LUTH and LASUTH) provide tertiary health care services to people living within and outside Lagos State. Lagos state has a population of over 20 million inhabitants [22].

### Duration of study

This study was completed within 16 months from the day of approval by the relevant authorities; an initial 12 months was approved in July 2020 by the Health Research Ethics committee of both institutions; however, due to the impact of COVID-19 on the clinical activity, the approval was extended by 6 months to ensure completion of the study.

### Study design

This is a cross-sectional study. All patients with histologically confirmed breast cancer undergoing chemotherapy that met the inclusion criteria were enrolled.

### Sampling method

All eligible and consenting patients at the clinics were consecutively recruited into the study until sample size was achieved.

### Sample size

The prevalence of CIPN in patients with breast cancer receiving oncological treatment in southwestern Nigeria could not be obtained with certainty; however, a study by Ezzi *et al* [13] in Kenya described the prevalence of CIPN with cisplatin as 83.6%. The minimum sample size is calculated using the formula [23]; *n* = Zα ^2^ P [1-P] / d2

*n* = Sample size for population more than 10,000

*Z* = Standard score

Where *Z*α = 1.96 at confidence interval of 95%

*P* = Maximum expected prevalence ratio % = 83.6%^13^

*D* = Margin of sampling error tolerated *d* = 0.05%

The minimum sample following a 20% Adjustment for attrition gave a sample size of 261.

### Eligibility criteria

#### Inclusion criteria

- Patients must have a histopathology report of breast cancer.
- Patients must consent to the study.
- All stages of breast cancer.
- Patients with a life expectancy of at least 6 months.
- Patients who received either neoadjuvant or adjuvant chemotherapy.

#### Exclusion criteria

- All patients without histopathological reports of breast cancer.
- Patients with recurrence who have received a neurotoxic chemotherapy previously as first-line treatment.
- Patients on medications that could cause peripheral neuropathy Disulfiram, Phenytoin, Amiodarone Hydralazine, Perhexiline, Nitrofurantoin, Isoniazid and Dapsone.
- All patients with uncontrolled Diabetes Mellitus and rheumatoid arthritis.

### Data collection procedure

All enrolled patients were counselled and assessed to ensure they met the inclusion criteria, and informed consent was also taken. All eligible participants were interviewed using a designed/semi-structured questionnaire administered by the interviewer on three occasions; before the commencement of chemotherapy, after completion of chemotherapy and 2 months after completion of chemotherapy to gather data on patient demographics and clinical information, which was retrieved from the participants’ medical records. Variables studied include; age, height, weight, body mass index (BMI), physical activity, stage of breast cancer, type of treatment (drug, dosage and frequency), dose delays, reductions or discontinuations. Information about the experience of CIPN symptoms and the onset of symptoms were collected using the European Organisation for Research and Treatment of Cancer (EORTC) CIPN20, time of onset of CIPN symptoms.

The EORTC CIPN20 contains 20 items assessing sensory (9 items), motor (8 items) and autonomic symptoms (3 items). However, 19 items will be used, as erectile dysfunction is not applicable to females. Using a four-point Likert scale (1 = ‘not at all,’ 2 = ‘a little,’ 3 = ‘quite a bit,’ and 4 = ‘very much’), individuals were to indicate the degree to which they have experienced sensory, motor and autonomic symptoms during the past week. Sensory raw scale scores range from 1 to 36, motor raw scale scores range from 1 to 32 and autonomic raw scale scores range from 1 to 12 for men and 1–8 for women (erectile function item is excluded) [24].

A limitation of the EORTC QLQ–CIPN20 is that a cut-off point indicating the presence or absence of CIPN is not yet established. However, a pilot study was conducted among 20 patients to validate the tool’s usefulness among the participants of the study. For this study, the maximum score was 76 (sensory, motor and autonomic) and the minimum score was 19. A score of 19 means No CIPN, a score of 20–39 is mild CIPN, a score of 40–57 is moderate CIPN and a score of 58–76 is severe CIPN. The EORTC QLQ–CIPN20 was used to characterise the severity and impact of CIPN, the presence or absence of CIPN was determined by a score >19.

### Statistical analysis

The collected data was imputed and analysed using a statistical package for social sciences. (IBM SPSS) version 25. Data was presented in proportions and frequency tables for categorical variables, while means and standard deviations were used to summarise continuous variables. CIPN was measured as a binary variable (yes/no). Numeric variables were presented as mean and standard deviation because they were normally distributed. The Kolmogorov–Smirnov test was used to assess data normality assumptions. The chi-square test and Fishers’ exact test were used to determine the association between independent categorical variables and CIPN status while the McNemar test was used to compare CIPN status at the end of chemotherapy and 2 months post chemotherapy. An independent student *t* test was used to compare mean quality of life according to CIPN status. Predictors of CIPN were determined using multiple logistic regression. Statistical significance was accepted as a *p*-value less than 0.05.

### Results

**Figure 1.**
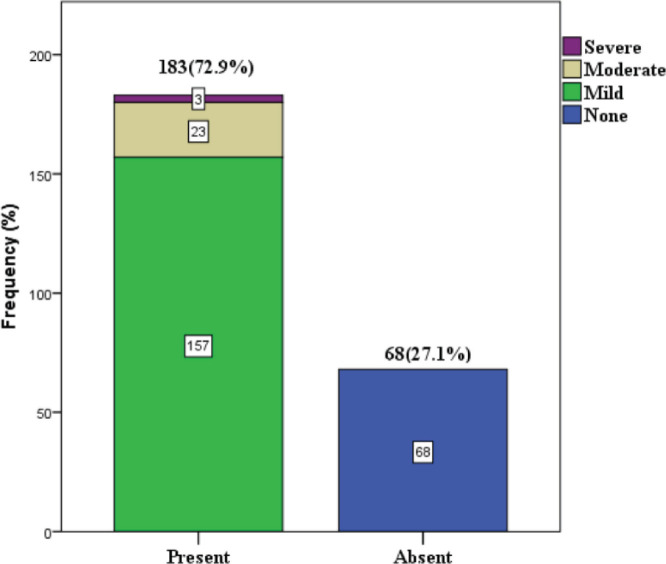
Prevalence and severity of CIPN.

A total of 261 participants were enrolled in the study, 10 of them were lost to follow up after baseline data was collected, hence they were excluded from the study. Thirty-one percent of participants were between 40 and 49 years old. The mean age of 49.48 +/- 11.4 years was reported, 49% had tertiary education, 76.1% were married and 38.6% had normal BMI (Table 1 and Figure 1).

**Figure 2.**
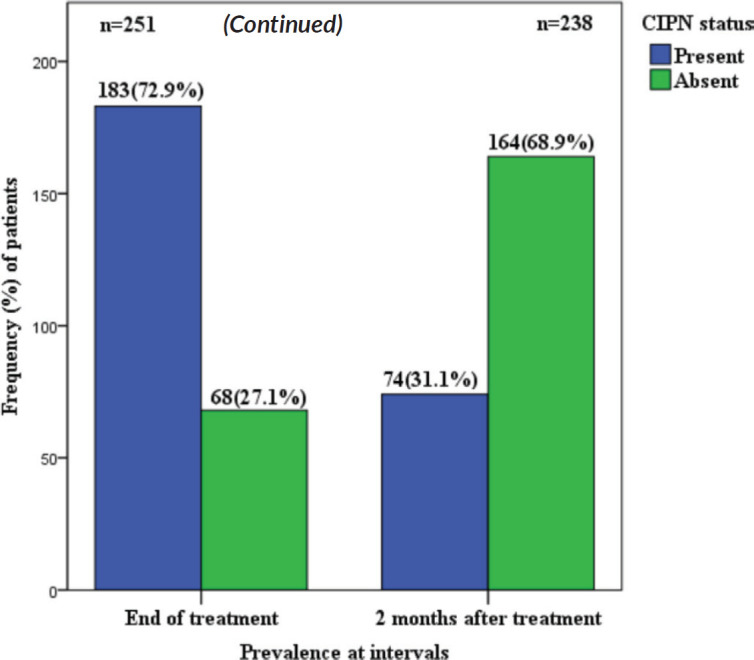
CIPN prevalence at end of chemotherapy and at 2 months post chemotherapy. The number of patients at 2 months after completion of chemotherapy has reduced to 238 because some of the patients were lost to follow up.

72.9% among the study participants reported symptoms of CIPN at the end of chemotherapy. One hundred and fifty seven (62.5%) had mild neuropathy, 23 (9.2%) had moderate neuropathy and 3 (1.2%) had severe neuropathy (Figure 2).

The prevalence of CIPN was 72.9% at the end of chemotherapy and 31.1% at 2 months post chemotherapy among the study participants who reported symptoms of CIPN (Tables 2 and 3).

## Discussion

CIPN is a common complication of chemotherapy. This study assessed the prevalence and predictors using validated instruments, among female breast cancer patients receiving varying regimens of chemotherapy in Lagos. Some of the findings of this study are consistent with the results of prior research, although comparison across studies of CIPN is limited by differences in study population, treatment regimens and assessment tools.

To the best of our knowledge, this is the first prospective cross-sectional study on the prevalence of CIPN in West Africa. It has provided a profile of CIPN among breast cancer patients treated with varying regimens used in breast cancer treatment. The mean age of the study population was 49.48 years, which is a relatively young population, this is consistent with a similar study done by Gaballah *et al* [25] in North Africa, where the mean age was 50.11 years, another study by Ezzi *et al* [13] in Kenya showed the mean age of 51 years among patients receiving cisplatin. A community practise in the United States showed a slightly higher mean age of 56.7 years [9].

The majority (74%) of the patients had locally advanced and metastatic disease (Table 4), this is a challenge in low and middle income countries (LMICs) where late presentation is common due to ineffective early detection programmes, low socioeconomic status and fear of mastectomy among others [26, 27], unlike the situation in the study by Simon *et al* [9] where about 79% of the participants were diagnosed with early breast cancer (stages I and II), though patients with metastatic disease were excluded from the study. In previous studies in Africa to assess the prevalence of CIPN among cancer patients, Gaballah *et al* [25] found the prevalence to be 46.8% in a retrospective analysis of 250 patients who were treated with varying types of chemotherapy, while Ezzi *et al* [13] found the prevalence in a cross-sectional study among patients receiving cisplatin chemotherapy to be 83%.

This current study found the prevalence rate of CIPN to be 72.9% at the end of chemotherapy (6 to 8 cycles), and 31.1% at 2 months follow up, of which most patients had mild to moderate peripheral neuropathy, this pattern of decline from 72.9% to 31.1% is near similar to the findings by Seretny *et al* [26] in a comprehensive review where the prevalence of CIPN was 68.1% when it was measured within the first month following chemotherapy, 60.0% at 3 months and 30.0% at 6 months or more.

The significant decline in the prevalence of CIPN throughout the study period is supported by the statistically significant reduction in the mean scores across the three assessed domains (sensory, motor and autonomic), as shown in Table 5. This is similar to the findings of a review by Argyriou *et al* [17] which examined studies on CIPN and observed that mild CIPN symptoms tend to improve or resolve within several months after the discontinuation of chemotherapy.

Our study also found that the majority (68.5%) of those who developed CIPN experienced only mild symptoms (Figure 1), which is consistent with the findings of Argyriou *et al* [17]. In another study, a similar observation with regard to severity was made among patients who received taxane, where 30% of participants rated their neuropathy symptoms as mild and 27.7 % rated their neuropathy as moderate and severe [9]. The most common symptom is numbness affecting the upper and lower limbs in which the majority (65.8%) were mild-moderate in severity, this is similar to the observation in a previous study [15] (Tables 6 and 5).

In this current study, it was observed that increasing age and lifestyle characteristics; alcohol intake and smoking have no significant association with CIPN, this is comparable to findings from other studies [19]. Although Bulls *et al* [28] showed in a study that older adults who develop CIPN are at higher risk of chronic CIPN, the mean age in this current study was lower (49.48 years). In another longitudinal study by Kleckner *et al* [29] which involved 200 women with non-metastatic breast cancer (aged 52 ± 10 years) receiving paclitaxel, it was found that patients who were more physically active before starting chemotherapy experienced less severe CIPN both immediately and 6 months after paclitaxel treatment. This contrasts with the findings of the current study, where physical activity scores were not associated with the development of CIPN.

The BMI is found not to be significantly associated with CIPN (Table 7), which is consistent with findings from another study by Simon *et al* [9] which shows no association between BMI and CIPN; however, it is at variance with findings in a study by Hershman *et al* [18] where CIPN was observed to be more among those who were overweight and obese compared to those who had normal weight.

The stage of the breast cancer was not found to be associated with CIPN (Table 7), this is unlike the observation in another study where CIPN motor impairment was marginally greater for patients with higher stages of breast cancer, specifically, stage III versus stages I and II [9]. There is a statistically significant association between cancer recurrence and CIPN in this study, this is not consistent with another study by Bao *et al* [30] where the recurrence of cancer was not significantly associated with CIPN.

There was no significant association between CIPN and the type of chemotherapy used for the patient, this is similar to findings from other studies [9, 31]; however, a study by Candeliero *et al* [32] where 229 patients with breast cancer who were treated with taxanes were retrospectively reviewed, it was observed that paclitaxel was more likely to cause CIPN compared to docetaxel. In this study, standard dose ranges of chemotherapy were used and there was no statistically significant association between the dose and CIPN. The participants received varying chemotherapy for their breast cancer due to the physician’s choice and the cost of the chemotherapy drugs (Table 8).

According to Table 9, sensory symptom scores of CIPN were significantly higher than motor and autonomic symptom scores among those who received AC then T, TAC, TP, AC/EC and FEC compared to the other combinations. A systematic review and meta-analysis of CIPN noted a similar observation as sensory symptoms were more common in comparison to motor and autonomic symptoms [12, 28]. There is no statistically significant correlation between CIPN and the molecular subtypes of breast cancer, this is different from the observations made in a study by Candeliero *et al* [32] where Her 2/Neu positivity is a statistically significant predictor of CIPN.

## Conclusion

CIPN is one of the most frequent side effects of chemotherapy. The prevalence of CIPN among breast cancer patients receiving combination chemotherapy was 72.9% at completion and the majority of these symptoms were mild. The prevalence reduced to 31.1% at 2 months after completion of chemotherapy.

Numbness in both hands and legs was the most common symptom reported among the participants. Age, stage of cancer, alcohol consumption, chemotherapy regimen and dose of chemotherapy were not found to be significantly associated with CIPN in this study, hence the need for further studies on genetic predisposition among Africans.

CIPN symptoms are very disturbing for patients and it impacts on their quality of life. It is important to provide health education and counselling on methods of reducing the severity of CIPN symptoms among patients including proper management of pre-existing medical conditions. Oral and topical agents available for the treatment of CIPN in our environment should be used for this condition.

## Recommendations

This study has shown the prevalence of CIPN among survivors or patients with breast cancer who received chemotherapy and how it impacts their quality of life. Hence, as physicians, it is important for us to not overlook it but to pay attention and be empathic the next time a patient reports a symptom of CIPN to ensure that the patient’s quality of life is improved significantly and that they are satisfied with our care.

## Limitations

1. The decrease in the sample size over cycles (some patients were lost to follow up, unreachable or died).
2. Hawthorne effect, where the participants modify an aspect of their behaviour in response to their awareness of being observed.
3. Objective measures such as neurological examination or nerve conduction studies were not carried out, as there is a lack of consensus on the value of these objective measures in daily practise and they do not always correspond to subjective complaints and influence on the quality of life.
4. We could not assess the immunohistochemistry in all participants as some of the participants are of low socioeconomic status and cannot afford the test, hence introducing a bias to its association with CIPN.
5. Some genetic predispositions (single nucleotide polymorphism) may be strong predictors of CIPN in the African population, hence further studies could be done in this area.
6. A wide variety of chemotherapy regimens used in this study may be responsible for the lack of association with CIPN, hence subsequent studies can be limited to smaller groups with balanced proportions receiving different chemotherapy regimens to allow for comparison.

## Conflicts of interest

There is no conflicts of interest.

## Funding

This study was self-funded.

## Consent to participate

Informed consent was obtained from all participants of this study by means of a consent form on which they appended their signature and kept a copy for themselves. A duplicate copy was kept with the researcher.

## Ethical approval

Ethical clearance to conduct this study was obtained from the Health Research Ethics committee of the LUTH and LASUTH, Ikeja.

## Figures and Tables

**Table 1.** Socio-demographic characteristics and clinical characteristics among participants.

Variable	Frequency (*n* = 251)	Percentage
Age group (Years) 20–29 30–39 40–49 50–59 60–69 ≥70 Mean ± SD	8 52 79 60 47 5 49.48 ± 11.4	3.2 20.7 31.5 23.9 18.7 2.0
Highest educational level None Primary Secondary Tertiary	8 25 95 123	3.2 100 37.8 49.0
Marital status Single Married Divorced Widowed	26 191 8 26	10.4 76.0 3.2 10.4
BMI class Underweight Normal Overweight Obese Mean ± SD	6 97 80 68 27.18 ± 5.5	2.4 38.6 31.9 27.1

**Table 2.** Predictors of CIPN.

Variable	Frequency (*n* = 251)	Percentage
History of smoking Current Previously Never	1 8 242	0.4 3.2 96.4
Alcohol consumption Yes No	62 189	24.7 75.3
Family history of neuropathy Yes No	2 249	0.8 99.2
Physical activity High Moderate Low	17 16 218	6.8 6.4 86.9
Drug regimen Doxorubicin + cyclophosphamide then paclitaxel Paclitaxel + doxorubicin + cyclophosphamide Paclitaxel + Capecitabine Paclitaxel + carboplatin Epirubicin or doxorubicin + cyclophosphamide Gemcitabine + cisplatin Docetaxel+ Herceptin then FEC + Herceptin FEC FEC then Paclitaxel + carboplatin Paclitaxel + carboplatin + Herceptin	25 44 3 16 111 3 9 34 4 2	10.0 17.5 1.2 6.4 44.2 1.2 3.6 13.5 1.6 0.8
Dose delays Yes No	10 241	4.0 99.6
Dose reduction Yes No	1 250	0.4 99.6
Discontinuation Yes No	2 249	0.8 99.2
Local/herbal therapy Yes No	48 203	19.1 80.9

FEC = 5-fluorouracil +epirubicin +cyclophosphamide

About 96.4% never smoked, 24.7% take alcohol, 99.2% have no family history of neuropathy over 80% had low physical activity, 44.2% of the patients received AC/EC, 19.1% of the patients had different forms of herbs while receiving chemotherapy

**Table 3.** Association between chemotherapy-induced neuropathy and the type of regimen.

	CIPN present (*n* = 183)	CIPN absent (*n* = 68)	*X* ^2^	*p*-value
Drug regimen Group I Group II Group III Group IV	53 (67.9) 25 (65.8) 87 (78.4) 18 (75.0)	25 (32.1) 13 (34.2) 24 (21.6) 6 (25.0)	3.681	0.298

FEC = 5- fluorouracil + epirubicin + cyclophosphamide

There is no significant association between CIPN and the chemotherapy regimen used. There was also no significant association even when the chemotherapy regimen was categorised into four groups

**Table 4.** Clinical characteristics among participants.

Variable	Frequency (*n* = 251)	Percentage
Site of disease among study participants Right Left Bilateral	103 133 14	41.4 53.0 5.6
Stage of disease I II III IV	4 61 108 78	1.6 24.3 43.0 31.1
Metastatic Yes No	78 173	31.1 68.9
*Metastatic location (*n* = 78) Liver Lung Bone Others	21 53 33 7	26.9 67.9 42.3 9.0
Histological type Invasive ductal carcinoma Invasive lobular carcinoma Invasive papillary carcinoma Mucinous carcinoma Others	220 5 5 6 15	87.6 2.0 2.0 2.4 6.0
Immunohistochemistry availability Triple negative Hormone receptor positive HER2 positive Triple positive Not available	80 41 16 4 110	56.7 29.1 11.3 2.8 43.8
Breast cancer surgery before chemotherapy Yes No	121 130	48.2 51.8
Type of breast cancer surgery (*n* = 121) Mastectomy Lumpectomy	88 33	72.7 27.2
Cancer reoccurrence (*n* = 121) Yes No	11 110	9.1 90.9

* Multiple location (patients have multiple metastatic sites)

Other sites of metastasis: spleen, vertebral spine

**Table 5.** Mean percentage CIPN scores at different intervals among participants.

	End ofchemotherapy	Two months post chemotherapy	*t*-value	*p*-value
Sensory	27.93 ± 6.4	22.85 ± 4.3	3.395	0.001*
Motor	27.53 ± 6.1	25.54 ± 5.8	2.031	0.032*
Autonomic	29.22 ± 11.1	27.12 ± 5.3	2.223	0.027*

(*) statistically significant

The mean scores in percentage in each domain over the interval among those who had symptoms of CIPN show a statistically significant decline in the sensory, motor and autonomic from the end of treatment to 2 months post treatment

**Table 6.** Severity of patient-reported symptoms of CIPN among participants.

Symptoms of CIPN (EORTC_CIPN20)	None	Mild (A little/Quite a bit)	Severe (Very much)
Tingling sensation in fingers, hands, toes or feet	155 (61.8)	89 (35.5)	**7 (2.8)**
Numbness in fingers, hands, toes or feet	92 (36.7)	**156 (62.2)**	3 (1.2)
Shooting or burning pain in fingers, hands, toes or feet	165 (65.7)	81 (32.3)	5 (2.0)
Cramps in hand or feet	166 (66.1)	84 (33.5)	1 (0.4)
Problems standing or walking because of difficulty feeling the ground under your feet	226 (90.0)	24 (9.6)	1 (0.4)
Difficulty distinguishing between hot and cold water	250 (99.6)	1 (0.4)	0 (0.0)
Problem holding a pen, which made writing difficult	244 (97.2)	6 (2.4)	1 (0.4)
Difficulty manipulating small objects with your fingers	242 (96.4)	9 (3.6)	0 (0.0)
Difficulty opening a jar or bottle because of weakness in your hands	233 (92.8)	17 (6.8)	1 (0.4)
Difficulty walking because your feet dropped downwards	236 (94.0)	14 (5.6)	1 (0.4)
Difficulty climbing stairs or getting up out of a chair because of weakness in your legs	125 (49.8)	**123 (49.0)**	3 (1.2)
Dizzy when standing up from a sitting or lying position	154 (61.4)	95 (37.8)	2 (0.8)
Blurry vision	205 (81.7)	46 (18.3)	0 (0.0)
Difficulty in hearing	249 (99.2)	2 (0.8)	0 (0.0)
Difficulty using the pedals while driving	242 (96.4)	8 (3.2)	1 (0.4)

Numbness in hands or feet (sensory) was the commonest symptom representing 62.2% of mild to moderate symptoms, followed by difficulty in climbing or getting up because of weakness in the legs (motor) with 49%. Tingling sensation in hands or feet has the highest frequency of severe symptoms at 7%

**Table 7.** Association between CIPN and socio-demographic, lifestyle and clinical characteristics.

Variable	CIPN present (*n* = 183)	CIPN absent (*n* = 68)	*X* ^2^	*p*-value
Age group (Years) 20–29 30–39 40–49 50–59 60–69 ≥70	6 (75.0) 40 (76.9) 56 (70.9) 42 (70.0) 37 (78.7) 2 (40.0)	2 (25.0) 12 (23.1) 23 (29.1) 18 (30.0) 10 (21.3) 3 (60.0)	4.409	0.492
BMI class Underweight Normal Overweight Obese	3 (50.0) 76 (78.4) 55 (68.8) 49 (72.1)	3 (50.0) 21 (21.6) 25 (31.3) 19 (27.9)	3.774	0.287
History of smoking Yes No	4 (33.0) 179 (74.0)	5 (55.6) 63 (26.0)	3.829	0.072
Alcohol consumption Yes No	42 (67.7) 141 (74.6)	20 (32.3) 48 (25.4)	1.113	0.291
Family history of neuropathy Yes No	0 (0.0) 183 (73.5)	2 (100.0) 66 (26.5)	5.426	0.020
Physical activity High Moderate Low	12 (70.6) 13 (81.3) 158 (72.5)	5 (29.4) 3 (18.8) 60 (27.5)	0.631	0.730
Primary site of disease Right Left Bilateral	76 (73.1) 95 (71.4) 12 (85.7)	28 (26.9) 38 (28.6) 2 (14.3)	1.311	0.519
Stage of disease I II III IV	3 (75.0) 40 (65.6) 83 (76.9) 57 (73.1)	1 (25.0) 21 (34.4) 25 (23.1) 21 (26.9)	2.522	0.471
Metastatic Yes No	58 (72.5) 125 (73.1)	22 (27.5) 46 (26.9)	0.010	0.921
Histological type Invasive ductal carcinoma Invasive lobular carcinoma Invasive papillary carcinoma Mucinous carcinoma Others	160 (72.7) 4 (80.0) 4 (80.0) 2 (33.3) 13 (86.7)	60 (27.3) 1 (20.0) 1 (20.0) 4 (66.7) 2 (13.3)	6.453	0.169
Breast cancer surgery before chemotherapy Yes No	88 (72.7) 95 (73.1)	33 (27.3) 35 (26.9)	0.004	0.950
Type of breast cancer surgery (*n* = 121) Mastectomy Lumpectomy	61 (69.3) 27 (81.8)	27 (30.7) 6 (18.2)	1.891	0.169
Cancer reoccurrence (*n* = 121) Yes No Molecular subtypes Triple negative Triple positive Hormone receptor positive HER 2 positive	11 (100) 77 (70) 57 (71.3) 3 (75.0) 27 (65.8) 12 (75.0)	0 (0.0) 33 (30.0) 23 (28.7) 1 (25.0) 14 (34.2) 4 (25.0)	4.538 1.898	0.0033* 0.754
Group I; Taxane + doxorubicin+ cyclophosphamide (TAC), Doxorubicin+ cyclophosphamide then Taxane (AC then T), Docetaxel+ Herceptin then FEC + Herceptin (TH then FECH). Group II; FEC Group III; doxorubicin or epirubicin + cyclophosphamide (AC/EC). Group IV; others: gemcitabine+ cisplatin (GP), paclitaxel + Capecitabine, paclitaxel + carboplatin (TP), FEC then paclitaxel + carboplatin (FEC then TP), paclitaxel + carboplatin+ Herceptin(TPH).				

**Table 8.** Association between the dose of chemotherapy and CIPN.

	CIPN present (*n* = 183)	CIPN absent (*n* = 68)	*p*-value
Doxorubicin + cyclophosphamide then paclitaxel			
50/500/175 mg^2^ 60/600/175 mg^2^	12 7	4 2	0.876
Paclitaxel + doxorubicin + cyclophosphamide			
175/50/500 mg^2^ 75/50/500 mg^2^	24 5	9 6	0.309
Paclitaxel/Capecitabine			
175/1000mg^2^	2	1	NA
Paclitaxel/carboplatin			
175/450 mg^2^ 75/60 mg^2^	12 2	2 0	0.568
Doxorubicin or epirubicin +cyclophosphamide			
60/600 mg^2^ 80/600 mg^2^	62 25	19 5	0.718
Gemcitabine +cisplatin			
1,000/60 mg^2^	1	2	NA
Docetaxel+ Herceptin then FEC + Herceptin			
100/600 mg^2^ then 600/90/600/600 mg^2^	5	4	NA
FEC/CAF			
500/50/500 mg^2^ 500/80/500 mg^2^	20 3	10 1	0.738
FEC then Paclitaxel + carboplatin			
500/80/500 mg^2^ then 175/450 mg^2^	2	2	NA
Paclitaxel + carboplatin + Herceptin			
175/450/600 mg^2^	1	1	NA

There is no significant association between varying doses of the different regimens of chemotherapy and CIPN

**Table 9.** Mean comparison of symptoms score according to drug regimen.

	Sensory	Motor	Autonomic	*p*-value
Doxorubicin + cyclophosphamide then paclitaxel	28.64 ± 6.2	27.88 ± 5.3	25.06 ± 4.9	<0.001*
Paclitaxel + doxorubicin + cyclophosphamide	30.74 ± 10.0	27.58 ± 7.7	26.30 ± 7.4	<0.001*
Paclitaxel/carboplatin	27.61 ± 5.2	27.39 ± 3.4	25.74 ± 3.0	<0.001*
Doxorubicin or epirubicin +cyclophosphamide	29.07 ± 7.4	27.73 ± 5.4	26.13 ± 5.7	<0.001*
Gemcitabine +cisplatin	25.00	25.00	25.00	0.894
Docetaxel+ Herceptin then FEC + Herceptin	26.54 ± 3.7	26.17 ± 2.3	25.00	0.103
Paclitaxel/Capecitabine	25.00	28.12	25.00	0.034*
FEC	28.68 ± 7.1	28.37 ± 6.8	26.04 ± 3.5	0.022*
FEC then Paclitaxel + carboplatin	25.00	25.00	25.00	0.402
Paclitaxel + carboplatin +	25.00	27.08 ± 1.8	25.00	0.03*

(*) statistically significant

Mean score of sensory symptoms of CIPN was significantly higher compared to motor and autonomic for those who received doxorubicin + cyclophosphamide than paclitaxel, Paclitaxel + doxorubicin + cyclophosphamide, Paclitaxel + carboplatin, Doxorubicin or epirubicin + cyclophosphamide and FEC
